# Application and progress of transcranial substantial ultrasound in Parkinson's disease

**DOI:** 10.3389/fneur.2022.1091895

**Published:** 2022-12-01

**Authors:** Xishun Ma, Tongxia Li, Lizhen Du, Tongliang Han

**Affiliations:** ^1^Department of Ultrasound, Qingdao Municipal Hospital, Qingdao, China; ^2^Department of Tuberculosis, Qingdao Chest Hospital, Qingdao Central Medical Group, Qingdao, China

**Keywords:** transcranial sonography, substantia nigra, Pakinson's disease, midbrain, hyperechogenicity

## Abstract

Parkinson's disease (PD) is a common nervous system disease, mainly manifested as motor retardation, resting tremor, etc. (1). The clinical features of early PD patients are not characteristic, and diagnosis is very difficult. When obvious PD manifestations are found, the number of dopaminergic neurons in substantia nigra of patients has been reduced by more than half, and the treatment is difficult (2). Early diagnosis or auxiliary diagnosis of PD in clinical work is crucial for the treatment of PD and the prognosis of patients. In recent years, cerebral ultrasound has been widely used in the diagnosis and treatment of some diseases, such as Parkinson's disease, Alzheimer's disease, tuberculous meningitis, brain injury, etc., especially for the study of PD. The European Union of neuroscience and the latest diagnostic guidelines for PD in China have confirmed the role of the transcranial sonography (TCS). This article reviews the recent advances in the study of PD by transcranial sonography.

## Introduction

PD is a common neurodegenerative disease in clinic. In recent years, its incidence rate is getting higher and younger (1). The gold standard for diagnosing PD is pathological diagnosis of brain tissue, but it has no significance for the diagnosis and treatment of PD in practical clinical work. At present, the diagnosis of PD is basically based on clinical symptoms, that is, motor symptoms and non-motor symptoms. Motor symptoms include postural balance disorder, motor retardation, myotonia and static tremor (2). Non motor symptoms mainly include mental symptoms, sleep disorders, anxiety and depression (3). The early symptoms of PD are not typical, and the clinical signs and symptoms overlap with other parkinson's disease syndromes, idiopathic tremor, and psychogenic dyskinesia, so the diagnosis of PD is indeed a great challenge (4).

At present, the pathogenesis of PD is not clear (5). Some studies have shown that the substantia nigra (SN) is one of the important structures in the pathophysiology of PD, and can even be considered as the only credible brain structure closely related to neuron loss (6). The loss of neurons and the lack of dopamine content in this mesencephalic nucleus usually lead to the occurrence of dyskinesia (7).

In 1995, Becker et al. (8) reported SN hyperechogenicity (SN+) of PD patients for the first time. Many studies have begun to invest in confirming the significance of TCS in the diagnosis and differential diagnosis of PD. 70~90% of PD patients showed hyperechoic substantia nigra (HSN) on TCS (9). At present, the latest European Neuroscience Union PD diagnostic guidelines and Chinese PD diagnostic standards (2016) (10) have recognized the auxiliary diagnostic value of TCS.

Compared with other examination methods, TCS has the advantages of non-invasive, convenient, low cost, high repeatability, non-radiation, and it is easier to be widely used in clinical work.

Then, we will elaborate on the application and progress of TSC in PD through the following aspects.

## The formation mechanism of SN+

Previous studies reported that iron deposition and microglial activation played an important role in the HSN (11). Zecca et al. (12) confirmed the viewpoint that HSN was associated with local iron deposition in the autopsy study. As a cofactor of tyrosine hydroxylase, iron is crucial to dopamine synthesis. Si et al. (13) considered that iron metabolism was related to PD motor symptoms. However, iron was found in the human body in a multi-medium form, and not all forms of iron deposition led to HSN. In some studies, ferritin injected into the substantia nigra of rats did not show HSN, but free Fe^3+^ injection caused HSN (14).

Some studies have reported that ceruloplasmin, as an iron oxidase, can oxidize ferrous ions to Fe^3+^, which can regulate the level of intracranial iron by promoting cell transport of iron ions. The decrease of ceruloplasmin activity may even lead to iron deposition (15), which can protect tissues from oxidative damage. Peripheral injection of ceruloplasmin can reduce neurodegeneration (16). Therefore, the study suggested that the decrease of ceruloplasmin level and the expression of HSN could play a role in the early iron deposition of PD patients.

The study found that the increase of ferritin content was consistent with the progress of PD. The increase of ferritin in early PD patients was mainly concentrated in melanin granules of dopaminergic neurons. The gray value of TCS can reflect the tissue density and ferritin content of ROI, and understand the changes of PD (17). At the same time, with the development of computer processing technology in the later stage, the quantitative analysis of ROI ultrasound gray value can effectively solve the problem of large subjective visual judgment error and improve the accuracy of early diagnosis.

The increase of hyperechoic area of substantia nigra was related to the degree of striatal system damage. With the aggravation of striatal system damage, the striatal dopaminergic system showed obvious abnormal changes, which was considered to be an important reason for the decline of intelligence and emotional disorder of PD patients (18).

## TCS operation method and SN+ determination method

**Figure 1:**
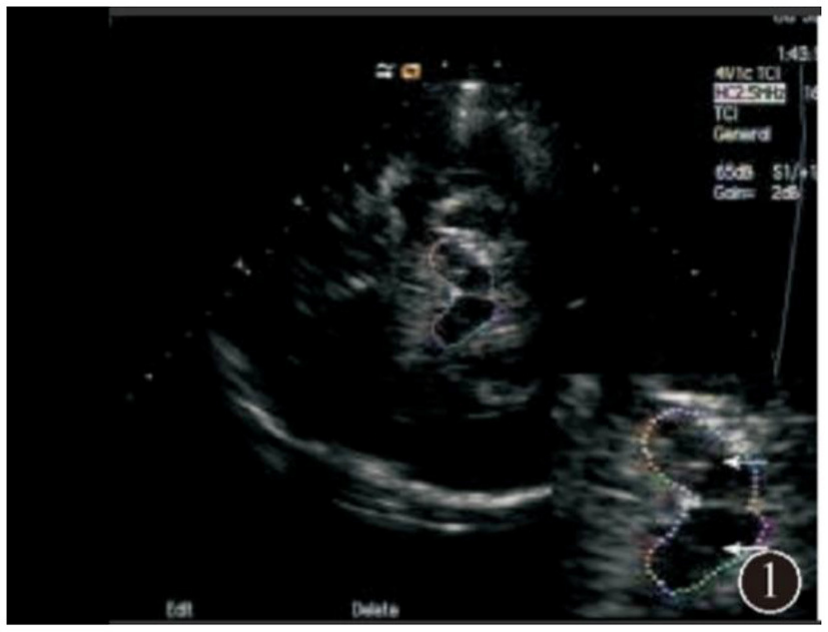
Normal substantia nigra echo.

**Figure 2:**
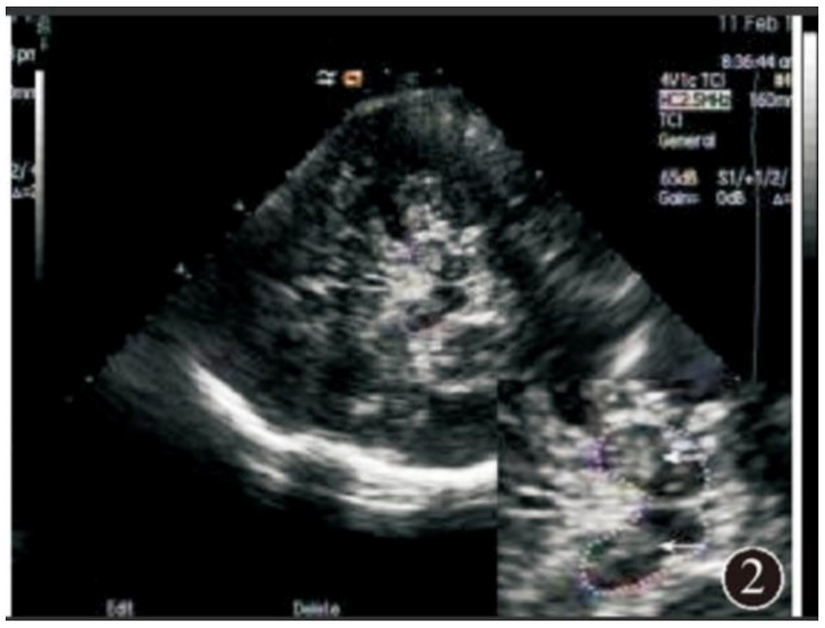
Abnormal substantia nigra hyperechogenicity.

Inspection method of TCS (19): The subject examined is in the supine position, and examiner presses the probe to the temporal window of the subject, parallel to the ear line (external auditory canal and eyes) scan brain horizontal slice, moving the probe near the temporal window to find the best sound window position to make the target image as clear as possible. Freezing the image, examiner manually draws the abnormal high echo signal to be measured with the cursor, and the instrument automatically calculates the area. In the midbrain region, the butterfly shaped midbrain is uniformly hypoechoic, the surrounding interpedullary cistern is hyperechoic, and the crescent shaped substantia nigra is uniformly hypoechoic. Specific cases (20) were shown Figures 1, 2.

At present, the indicators for TCS to determine SN+ in PD patients include qualitative and quantitative indicators. The qualitative indicators mainly adopt the classification method proposed by Bartova et al. (21): Grade I: Uniformly distributed low echo; Grade II: Scattered dot and thin line slightly high echo can be seen; Grade III: The echo is patchy and lower than the echo of the interpedullary cistern; Grade IV: The echo is patchy and equal to the echo of the interpedullary cistern; Grade V: The echo is patchy and higher than the echo of the interpedullary cistern.

There are two commonly used quantitative indicators, one is the area of SN+, and the other is the ratio of bilateral total area of SN+ to total area of midbrain (S/M). Huang et al. (22) considered that the ratio ≥7% was positive, and the area of SN+ was mainly 0.18~0.25 cm^2^ (23).

## SN+ characteristics and influencing factors of PD patients

### Gender

Some studies showed that the reason for the higher male proportion in the PDSN+ group may involve the epidemiological characteristics of PD, which could be related to the higher prevalence of male PD. Another explanation was that it was affected by estrogen. Related studies (24) found that estrogen could reduce the iron level in the body, making it harder for women to accumulate iron.

### Non-motor symptom characteristics

PD patients were found with SN+ to be in a later H–Y (Hoehn and Yahr scale) stage. One study (25) found that iron may be able to induce dopaminergic neurodegeneration through neuroinflammatory mechanism, mediated by microglia hyperactivation and neurotoxic factors, and microglial activation can drive disease progression, which may lead to SN+ PD patients in late H–Y stage, and its disease progressed rapidly. However, in earlier H–Y stage of PD patients, this effect may not be obvious. The research results showed that the PDSN+ group had lower scores on the MoCA score (Montreal Cognitive Assessment Form) than the PDSN– group, indicating that the PDSN+ group had more severe cognitive impairment. Non-motor symptoms of PD have a complex pathogenesis, and there are two pathways in the brain thought to be related to cognitive function, namely the meso-limbic pathway and a cortical pathway (26) in the meso-limbic system. Among them, the meso-limbic system-cortical pathway also contains dopamine neurons. If it is damaged, the dopamine contents will decrease, which can lead to damage in cognitive function such as memory. Thus, the depletion of dopamine triggers an enhanced echogenicity in the substantia nigra, and the PD patients with SN+ show a more severe cognitive dysfunction.

### Stability

The relationship between SN echo and apoptosis of dopaminergic neurons is very complex, which does not reflect the degree of apoptosis or the number of remaining dopaminergic neurons, and may only be a relatively stable signal of substantia nigra lesions earlier in the disease. In some follow-up study (27) lasting 3 or even 8 years, SN echo remained relatively stable during the course of the disease, and failed to detect a significant change in echo grade even with two interval measurements. These results suggested that disease duration could not be a determinant factor in iron deposition in the substantia nigra and associated abnormal echo enhancement in PD patients.

### Asymmetry

Studies showed that PD was usually an asymmetric disease (28). Some reports found that SN+ in PD patients was unevenly distributed between the left and right sides, and that the left SN+ area was almost always greater than that in the right (29), which may be the reason for more dopamine metabolism in the left SN than on the right side (30). Kempster et al. (31) found a more significant loss of dopaminergic neurons in initiating SN+ than in non-initiating SN+.

### The value of SN+ in the diagnosis of PD

In recent years, TCS has been widely applied to neurological diseases. Some studies found that TCS could reflect the specific physiological and pathological processes of substantia nigra in PD patients. SN+ was an early marker of nigrostriatal damage, and it was a susceptibility risk factor for PD, so the significance of substantia nigra hyperechogenicity in PD had been gradually recognized (32).

With the deepening of research, it was found that in the early stage of PD, non-motor symptoms would appear, mainly including the rapid eye movement period of behavior disorders, olfactory disorders, and autonomic symptoms. Therefore, finding clinical markers of non-motor symptoms was particularly important when Parkinson was in its early stage (33). In the study, when transcranial ultrasound combined with non-motor symptoms evaluation to diagnose Parkinson's disease, the diagnostic sensitivity, specificity and positive predictive value improved, indicating that the diagnostic accuracy increased (34).

Studies showed that hyperechogenicity of the substantia nigra can reflect the severity of the disease, and it was somewhat associated with the duration of the disease and the age of patients (35). In the early stage, hyperechogenicity indicated the primary PD, and it suggested an atypical PD if it appeared hypoechoic in the substantia nigra or with a hyperechoic biconvex nucleus (36). Therefore, the TCS technology can play an important role in monitoring the high-risk PD population in real time.

Some studies showed that the ratio of substantia nigra hyperechogenic area and midbrain area (S/M) can reflect the ROI cell arrangement and structure state. The increased ratio indicated that the abnormal cell function continued, the number of cell necrosis and apoptosis increased, and the risk of softening and calcification increased. Therefore, with the longer duration of patients, the ratio of S/M gradually increased, and the neurological damage further aggravated (28).

One study (37), the subjects were divided into PD patients HSN group, NSN group and healthy control group. The level of ceruloplasmin was lower in the HSN group and in the NSN group than in the control group, and the proportion of patients with ceruloplasmin levels below the normal range was higher in the HSN group than in the NSN group. Transferrin levels were inversely associated with HSN area. Decreased ceruloplasmin levels may be a factor contributing to the development of HSN in PD patients, and the HSN area was inversely correlated with transferrin levels.

A previous study showed that using the hypechoic area of ≥0.20 cm^2^ in substantia nigra as the gold standard for diagnosis, the specificity of TCS diagnosis was 83.4%, a sensitivity of 97.7%, a positive predictive value of 92.9%, and an accuracy of 88.3% (38). There were studies found that prolonged T2 signal was seen (39) on head MRI in people with enhanced echogenicity, which indicated that enhanced echogenicity of SN may be related to iron content. Riederer et al. (40) found that increased iron levels early in the course of PD were initially localized to melanin granules in DA ergic neurons, and that increased ferritin levels were consistent with the progression of the course of PD disease. Therefore, it was suggested that echogenic change in substantia nigra was mainly related to (41) with changes in tissue iron and neuromelanin content.

## The value of SN+ in the differential diagnosis of PD

### Identification of PD and essential tremor (ET)

Previous studies showed that TCS played an important role in the differential diagnosis of PD and ET, with its sensitivity, specificity, and positive predictive values of 75~86, 84~93, and 91~95% (42), respectively. After a systematic review and meta-analysis of the differential diagnosis of SN+ for PD with TCS, Shafieesabet et al. (43) concluded that SN+ had 75% sensitivity and 70% specificity in distinguishing PD from atypical parkinson's syndrome. The sensitivity and specificity in identifying PD from essential tremor were 78 and 85%, respectively, and although the sensitivity and specificity didn't meet the optimal standard, the TCS examination could identify PD from motor disorder diseases with similar symptoms, especially for the identification of atypical parkinson's syndrome.

### Identification of PD and Parkinson's superposition syndrome

Walter et al. (9) found that using TCS to detect SN+ played an important role in the early differential diagnosis of PD and Parkinson's superposition syndrome with sensitivity, specificity, positive predictive value, negative predictive value and diagnostic accuracy were 94.8, 90, 97.4, 81.8, and 93.9%, respectively.

Another study found that the sensitivity, specificity, positive predictive value, negative predictive value and diagnostic accuracy of hyperechogenicity in lenticular nucleus under TCS for early differential diagnosis of PD and Parkinson's syndrome were 66.7, 68.6, 35.3, 88.9, and 68.2%, respectively (44).

### Identification of PD and vascular Parkinson's syndrome (VP)

Venegas Franke (45) reviewed that compared with PD patients, most VP patients had normal echo in the substantia nigra of the midbrain. Compared with patients with Parkinson's Superposition Syndrome, the echo of basal ganglia in VP patients was also normal. In addition, TCS could also monitor the degree of vascular stenosis in patients. Combined with the above indicators, TCS had a high specificity in the diagnosis of VP, so TCS played an important role in the differential diagnosis of PD and VP.

### Differential identification between PD and depression

Usually SN+ was more common in PD patients, but not in depressed patients. If the SN of PD patients was hyperechogenicity and was accompanied by depressive symptoms, the depressive symptoms can be regarded as a concomitant symptom of PD or a non-motor symptom (46) in the early stage of PD.

## Limitations

The clinical application of TCS also has limitations: (1) some subjects have too small temporal windows, and TCS cannot detect clear midbrain echo. The penetration of temporal window is affected by age, gender, and race. Somen studies showed that 15–30% of Asians cannot detect nigra due to poor temporal window penetration, especially in elderly women. Because of the decreased estrogen secretion after menopause, poor temporal window penetration was more common than male patients of the same age (47); (2) TCS depends on the proficiency of operation and diagnostic physicians, and has a subjective tendency; and (3) the standard is not yet unified.

## Future and challenge

In the future, we hope that the ultrasound equipment can be more refined, so as to make up for the disadvantage that some subjects cannot carry out normal examination. During various parameter measurement processes, it is hoped that AI technology can be used to reduce the error of manual measurement. Finally, it is hoped that clinical workers will conduct more clinical research, so as to develop unified TCS standards, and better apply them in the diagnosis and treatment of PD.

## Author contributions

All authors listed have made a substantial, direct, and intellectual contribution to the work and approved it for publication. XM was mainly responsible for writing the paper. TL and TH were responsible for organizing the ideas of the paper and provided opinions. LD was responsible for checking the details of the paper and correcting some mistakes.

## Funding

This work was supported by grants from Qingdao Medical Research Guidance Plan of 2020 (No. WJZD018).

## Conflict of interest

The authors declare that the research was conducted in the absence of any commercial or financial relationships that could be construed as a potential conflict of interest.

## Publisher's note

All claims expressed in this article are solely those of the authors and do not necessarily represent those of their affiliated organizations, or those of the publisher, the editors and the reviewers. Any product that may be evaluated in this article, or claim that may be made by its manufacturer, is not guaranteed or endorsed by the publisher.
